# A Wearable Strain Sensor Utilizing Shape Memory Polymer/Carbon Nanotube Composites Measuring Respiration Movements

**DOI:** 10.3390/polym16030373

**Published:** 2024-01-29

**Authors:** TranThuyNga Truong, Jooyong Kim

**Affiliations:** 1 Department of Smart Wearables Engineering, Soongsil University, Seoul 156-743, Republic of Korea; thuynga290391@gmail.com; 2 Department of Materials Science and Engineering, Soongsil University, Seoul 156-743, Republic of Korea

**Keywords:** resistive stretch sensor, e-textiles, wearable sensor, flexible sensor, respiration sensor

## Abstract

Flexible wearable sensors are integral in diverse applications, particularly in healthcare and human–computer interaction systems. This paper introduces a resistive stretch sensor crafted from shape memory polymers (SMP) blended with carbon nanotubes (CNTs) and coated with silver paste. Initially, the sensor’s characteristics underwent evaluation using a Universal Testing Machine (UTM) and an LCR meter. These sensors showcased exceptional sensitivity, boasting a gauge factor of up to 20 at 5% strain, making them adept at detecting subtle movements or stimuli. Subsequently, the study conducted a comparison between SMP-CNT conductors with and without the silver coating layer. The durability of the sensors was validated through 1000 cycles of stretching at 4% ∆R/*R*_0_. Lastly, the sensors were utilized for monitoring respiration and measuring human breathing. Fourier transform and power spectrum density (PSD) analysis were employed to discern frequency components. Positioned between the chest and abdominal wall for contact-based respiration monitoring, the sensors revealed a dominant frequency of approximately 0.35 Hz. Signal filtering further enhanced their ability to capture respiration signals, establishing them as valuable tools for next-generation personalized healthcare applications.

## 1. Introduction

Recently, wearable sensors integrated into textiles have received significant interest in personalized healthcare applications [1], including for the measurement of heart rate, body temperature [2], and respiration rate [3]. Many wearable sensors have been developed effectively by embedding conductive fillers like graphene, carbon nanotubes, and conductive polymers into polymer films or by integrating semiconductors into flexible thin metal substrates [4,5]. In particular, the ability of wearable, flexible, and stretchable strain sensors to transform mechanical deformations into electrical signals has recently received much interest [6]. These sensors are widely used in soft robotics [7], human–machine interfaces [8], health monitoring systems [9], and human motion tracking and detection [8,10,11]. Based on modifications in electrical properties in response to mechanical deformations, there are many types of stretchable strain sensors, such as resistive sensors [12,13,14,15], capacitive sensors [16], and piezoelectric sensors [17]. Among many stretchable sensors, resistive strain sensors with high sensitivity are highly popular due to their typical construction and simple manufacturing process.

To achieve heightened sensitivity, the microstructures of conductive materials in stretchable resistive strain sensors must undergo significant alterations in resistance in response to subtle stimuli. Recently, there has been a proposal to harness the capabilities of graphene, carbon nanotubes (CNTs), and additional materials in the development of stretchable resistive strain sensors, aiming to enhance their flexibility and sensitivity [18,19,20]. Carbon nanomaterials possess attractive characteristics, including remarkable flexibility, a substantial surface-to-volume ratio, excellent chemical and thermal stability, and strong electrical conductivity. 

Their remarkable conductivity notwithstanding, such sensors lack stretchability or show minimal stretching when used in high volume ratios, which greatly restricts their capacity to detect human motion. Additionally, wearable strain sensors must be highly durable for human motion detection; to do that, the substrate materials must be highly adaptable to achieve long-term integration with the human body for physiological signal monitoring. Additionally, due to the unavoidable degradation from mechanical damage and repeated use, the rapid healable capacity is essential for extending the life and durability of wearable strain sensors. It is worth noting that research indicates that the comfort range for human skin falls between 30 and 34 °C. The relatively low thermal conductivity of human skin makes a potential for heat buildup at the interfaces between sensors and the skin. To address this concern, a material that is affected by an external stimulus such as temperature change, like shape memory polymer that can be reshaped and temporarily set before returning to its original shape upon the removal of the external stimuli, becomes essential.

This paper introduces a flexible, breathable, and biocompatible strain sensor equipped with advanced thermal management capabilities designed for the purpose of measuring respiration. There are two different ways to measure respiration: contact methods, in which an instrument is attached to the subject’s body, and noncontact methods, in which the equipment is not. Although noncontact has the advantage of convenience, it is highly technical and complex in terms of signal processing and fabrication. Additionally, due to their limitations, a large number of technologies have not yet been broadly embraced in general healthcare. For example, signals from radar, optical, and thermal sensors can also be used to infer the respiratory rate indirectly [21,22]. Although versatile in application, these noncontact methods face challenges such as accuracy issues arising from environmental noise, particularly in radar signals. The diminished transmitted power in free space makes these signals susceptible to interference and loss, especially in the presence of internal factors like patient body movements. The contact method, including a belt on the chest or abdomen, on the other hand, offers enhanced accuracy. Significantly, mercury strain gauges or impedance techniques are the best ways to quantify the movements of the chest and abdominal walls [23]. Despite advancements in signal processing, the contact method remains superior in accuracy, overcoming the challenges associated with noise and environmental interference present in noncontact methods.

In this research, we have developed a high-sensitivity strain sensor using a combination of shape memory polymers (SMPs) and carbon fibers (CNT/SMP), which is then coated with silver ink. The wearable strain sensor is based on the idea that the resistance alters in accordance with stretching to detect respiration movement. This sensor comprises a highly conductive layer (silver layer) applied onto a stretchable and moderately conductive coil (single-walled CNT/shape memory polymer). The core emphasis of our investigation lies in the comprehensive evaluation of the sensing capabilities and mechanical characteristics of the SMP when enhanced through physical blending with CNTs. Our developed strain sensor showcases high sensitivity, achieving up to 120% responsiveness when subjected to a 5% strain. Furthermore, we propose the use of this strain sensor for wearable applications, where changes in resistance due to stretching can be employed to detect respiratory movements. This concept holds promise for lightweight, cost-effective, and market-friendly respiration monitoring devices. With its high sensitivity and adaptability, our strain sensor presents a compelling solution for advancing the field of wearable technology in the realm of respiratory monitoring.

## 2. Experimental Section

In this section, we will briefly introduce how to fabricate the respiration sensors mentioned in the previous section in the following order. Firstly, we show how to generate SMP-SWCNT composites using acid treatment to make a conductor and then coating it with silver paste. Finally, this section focuses on manufacturing a respiration sensor that is highly sensitive in the detection of respiration movement. 

### 2.1. Fabrication of SMP-mSWCNT Pellets

An acid treatment process was employed to enhance the dispersion of carbon nanotubes (CNTs) within the composite material. This treatment introduced structural cracks in the carbon–carbon bonds of the CNTs, increasing their surface modification and thereby improving the electrical conductivity of the resulting composites. The addition of functional groups to the CNT surface through acid functionalization caused these bundles to separate due to repulsive forces, leading to better dispersibility in polar solvents [24,25,26].

**Figure 1:**
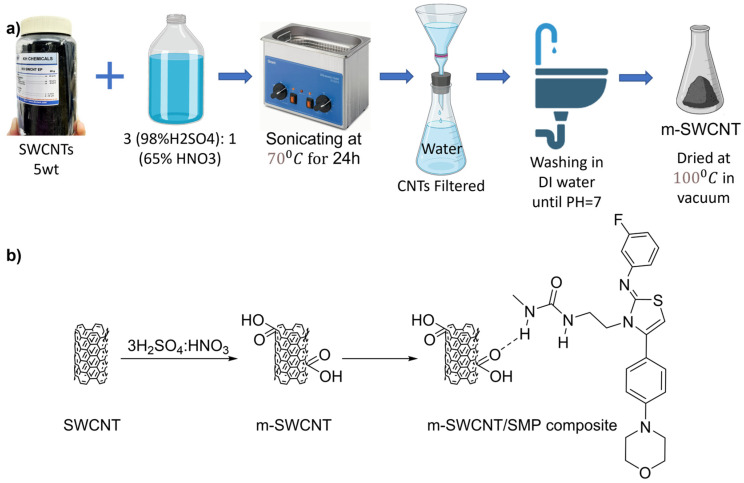
(**a**) Purification of SWCNT using acid treatment; (**b**) representation of the hydrogen contact that is most likely to occur.

In the experiments, ultrapure HNO_3_ 65% and H_2_SO_4_ 98% (*w*/*w*) from KH Chemicals in Korea were used. As depicted in Figure 1a, single-walled carbon nanotubes (SWCNTs) with dimensions of 1–1.4 nm in diameter and 5–50 μm in length, also sourced from KH Chemicals in Korea, were initially cleaned in deionized (DI) water or distilled water before undergoing treatment with two different acid concentrations: a 3:1 solution of concentrated H_2_SO_4_ (98%) to HNO_3_ (65%). This treatment involved sonicating the SWCNTs in a water bath at 70 °C for 24 h. The acid treatment introduced -OH groups on the SWCNT surface, forming hydrogen bonds with the water molecules, as shown in Figure 1b. Subsequently, the SWCNTs were neutralized by rinsing them with DI water or distilled water, collected on a polytetrafluoroethylene filter, and washed again with water until they reached a neutral pH. Finally, the modified SWCNTs were dried at 100 °C in a vacuum oven.

The shape recovery rate of CNT/SMP composites can be improved depending on the heat treatment and the content of CNTs inside the polymer [27,28]. The composite may have had some internal residual stresses or strains in the interface between the polymer and the CNTs; this could explain the improved electrical conductivity and reduced flexibility. Therefore, the CNT volume fraction plays an important role in the mechanical characterization of conductors. After the experimental process, the suitable volume addition of CNTs into the polymer was around 1.2%. The fabrication of CNTs-SMP pellets prepared for creating SMP/CNT filaments is represented below.

**Figure 2:**
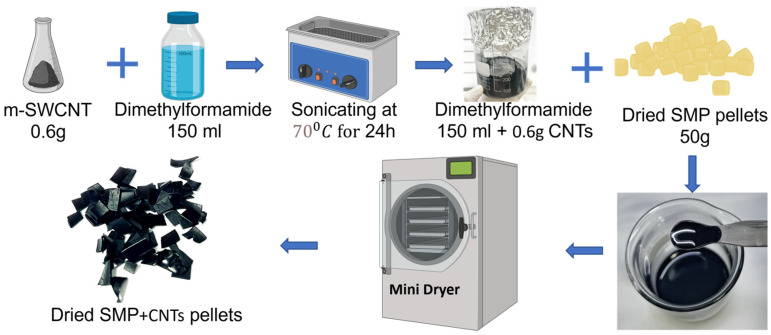
Fabrication process of SMP/CNT pellets.

As shown in Figure 2, using a sonicator at 70 °C for 24 h, the SWCNT was dispersed in dimethylformamide (DMF; from DAIHAN Scientific Co., Seoul, Republic of Korea). The SMP pellets (polyurethane, MM2520-from SMP Technologies Inc., Tokyo, Japan) with a glass transition temperature ($T_{g}$) of 25 °C were dried at 90 °C for four hours in a vacuum oven to remove moisture before melting. A magnetic stirrer was utilized to dissolve the shape memory polymer (SMP) in dimethylformamide (DMF) at 170 °C. The composite materials underwent a sequential heating and cooling process. After 48 h at 170 °C, the m-SWCNT/DMF and SMP/DMF solutions were mixed and stirred. The m-SWCNTs were evenly dispersed within the SMP matrix, primarily due to hydrogen bonding interactions between the carboxyl (-COOH) groups of the m-SWCNTs and the urethane (-NHCO-) groups of the SMP. The carboxylic acid functional groups on the m-SWCNTs facilitated strong interfacial adhesion to the surrounding matrix [29]. Subsequently, a knife-coating device (KP-3000, Seoul, Republic of Korea) from Kipae Co. was employed to create films of SMP/CNTs. These films were then placed in a vacuum and dried at 100 °C to eliminate any residual DMF solution. The dried SMP/CNT films were subsequently cut into pieces, as depicted in Figure 2, for the subsequent steps in the process.

### 2.2. Preparation of Respiration Sensors

**Figure 3:**
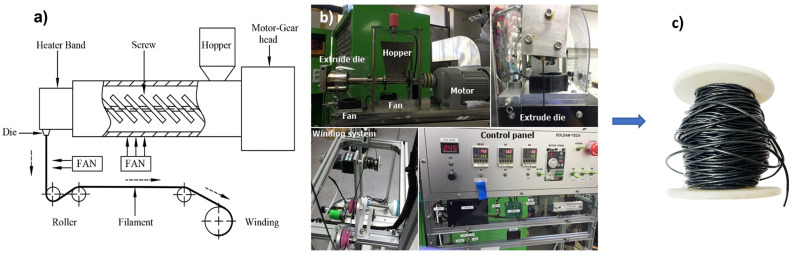
Filament production. (**a**) Schematic diagram of extrusion system; (**b**) extrusion system; (**c**) SMP/CNT filament roll.

**Figure 4:**
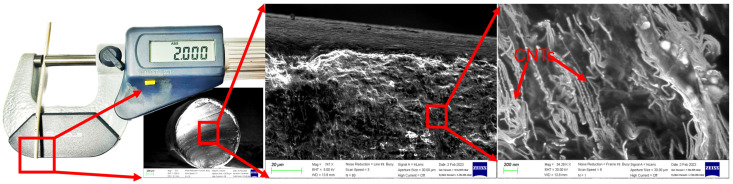
SEM of respiration sensor.

The dried SMP/CNT pellets were put into the extruder’s hopper, as can be seen in Figure 3. The extruder used a plasticator consisting of a rotating spiral screw inside a heated barrel to melt the pellets. The melted composite was pressed through a 2 mm diameter die to produce a suitable continuous product shape. The extrusion temperature was set at 145 °C. The resulting filaments had a diameter of 2 mm with a variation of around 0.05 mm, as can be seen in Figure 4. After that, the conductor was coated with a silver paste to increase sensitivity and detect respiration movement. The characteristics and curing conditions of the silver paste from KH Chemicals in Korea utilized in this study are shown in Table 1. After coating with silver, the sensors were placed inside a mini dryer and cured at 100 °C for 10 min to remove the solvent.

Raising the temperature beyond 100 °C results in a reduction in resistance, but it also leads to the formation of cracks on the surface silver layer as nearly all solvents are removed, diminishing the sample’s stretchability [26,30]. Therefore, the samples coated with silver paste were cured at temperatures ranging from approximately 60 to 100 °C to strike a balance between resistance reduction and maintaining optimal stretchability. This temperature range allowed the samples to retain a remarkable stretchability of up to 140%. The structural and surface characteristics of the single-walled carbon nanotubes (SWCNTs) were examined using a scanning electron microscope (Gemini SEM 300 from ZEISS, Oberkochen, Germany). Figure 4 reveals that the diameter of the modified SWCNTs (m-SWCNTs) falls within the range of 1 to 1.5 nm. The bonding between SWCNTs and the shape memory polymer (SMP) was enhanced post-modification, primarily because the m-SWCNTs featured carboxyl (-COOH) functional groups attached to their ends and sidewalls. Furthermore, the SEM images in Figure 4 clearly illustrate the interconnected nature of the SMPs and CNTs. The re-aggregation of CNTs within the polymer matrix was effectively prevented due to the formation of robust covalent bonds between the CNTs and SMPs.

### 2.3. Characterizations

**Figure 5:**
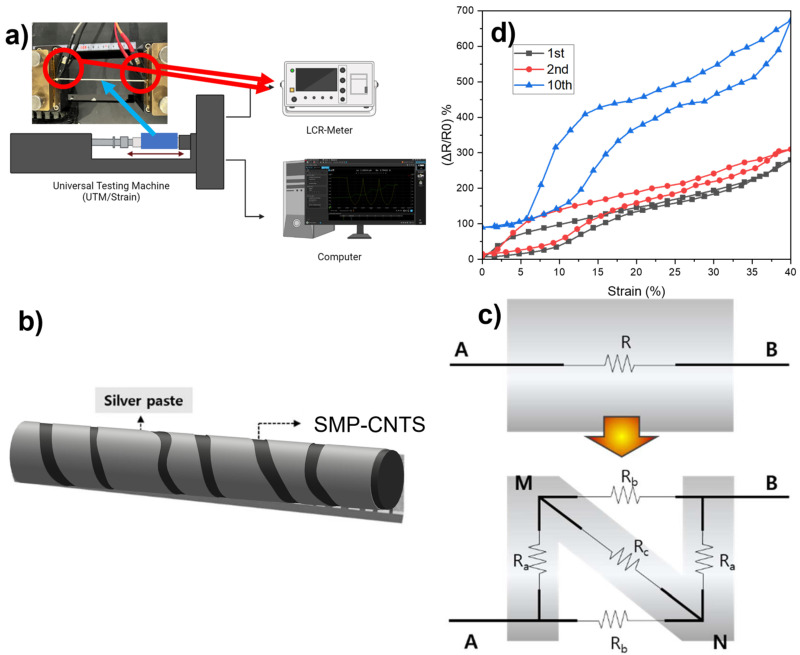
(**a**) Schematic diagram of experiment measurement; (**b**) schematic structure of the sensor after strain; (**c**) resistance model of the crack structure; (**d**) stretch/release sensing properties at the first (1st), second (2nd), and tenth (10th) stretch.

The schematic diagram of experimental measurement for evaluating the performance of the proposed sensors is shown in Figure 5a. A Keysight LCR meter (E4980AL) and a Universal Testing Machine (Dacell Co., Seoul, Republic of Korea) were employed to carry out the resistance test under the different strain powers. R_0_ is the sensor’s initial resistance when it is pre-stretched, and ∆R is the absolute change in resistance under the straining process. These values were used to determine the relative resistance changes (∆R/$R_{0}$).

## 3. Results and Discussion

### 3.1. Enhancement of Sensitivity through the Introduction of Cracks

The strain sensor, which comprises a composite structure of SMP mixed with SWCNTs below and a silver top layer, is schematically depicted after stretching in Figure 5b. The fractures expanded during elongation and adjusted themselves after the sensor was released. The strain sensor’s operating principle under stretching is illustrated in Figure 5c, and it significantly depends on the dimensional changes in the crack structures along the sensor’s surface. Equations (1) and (2) can be used to compute the resistance of the sensor construction before and after the constructed cracks were produced, respectively [31].

(1) $$R_{AB(initial)}=R_{silver\;layer(initial)}//R_{SMP-CNTs(initial)}$$

where $R_{silver\;layer(initial)}$ and $R_{SMP-CNTs(initial)}$ represent the resistance of the silver-layer-coated composite and the resistance of the composite without the silver layer, respectively. Note that, due to the low mass fraction of CNTs inside the composite, the resistance of the composite is huge, while the resistance of the silver layer is small. As a result, the resistance of the sensor $\left(R_{AB\left(initial\right)}\right)$ is approximately equal to the resistance of the silver layer ($R_{silver\;layer(initial)}$). This mechanism could provide a more in-depth understanding of the resistance exhibited by the crack structure using a simplified model derived from [32].

(2) $$R_{AB(after)}=\frac{R_{a}R_{c}+2R_{a}R_{b}+R_{b}R_{c}}{R_{a}+2R_{c}+R_{b}}$$

where $R_{a}$, $R_{b},R_{c}$ are the resistance of each segment AM, BN, and MN, respectively. According to [31], the adjacent CNTs become increasingly separated as the strain rises. $R_{a}$, $R_{b},\mathrm{and}\;R_{c}$ should all gradually rise, which causes the resistance after cracking ($R_{AB(after)}$) to rise as well.

**Figure 6:**
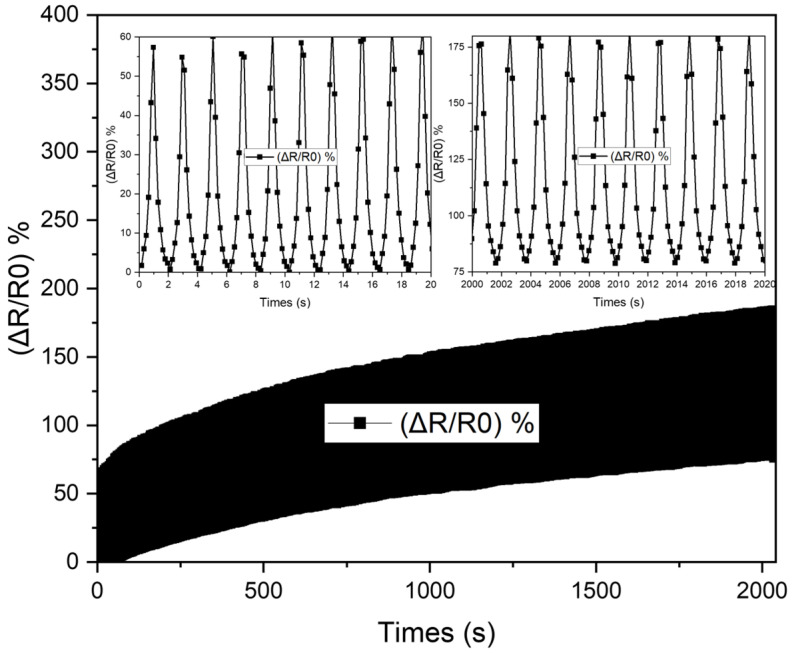
Durability after 1000 stretching and releasing cycles at 4% stretch.

**Figure 7:**
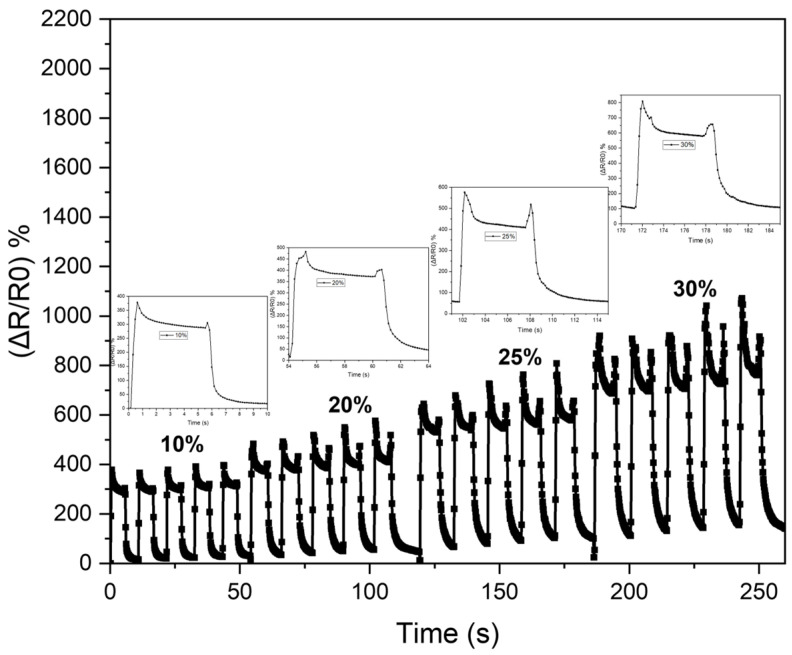
Hysteresis at various stretch levels.

Figure 5d shows the stretch/release sensing properties at the 1st, 2nd, and 10th stretches. A significant hysteresis curve exists between the first and tenth cycles. A significant hysteresis curve exists between the first and tenth cycles when applying a strain exceeding 40%. Conversely, this effect demonstrates minimal variation when the strain is less than 10%, as depicted in Figure 6. The variation between the second and first cycles is minimal, though. A strain sensor detects resistance deviations caused by changes in conductive pathways when a mechanical strain is applied, attributed to the electron tunneling that develops between neighboring conducting components [33,34]. As cracks develop under stretching, reducing the contact between the silver and SMP-CNTs coil, these conductive routes suffer more significant harm. In the process of extending, the electrical resistance gradually rises. When a sample is released, the elongation-induced gaps become smaller, the disparate regions are linked, the conductive pathways are restored, and the resistance gradually falls [35,36]. Herein, the interaction between the stretchy layer (SMP-CNTs) and the silver paste coating results in such geometrical variations in the structural fractures. Due to the silver paste coating’s far better conductivity than that of SMP-CNTs, the pre-cracked strain sensor has low initial resistance. A low starting resistance brings two benefits: achieving higher sensitivity and increasing the shape recovery ratio. Combining CNTs with SMP makes the sensor conductive, but the resistance is still very high [20]. As we know, the shape memory polymer needs a temperature higher than the transition temperature ($T_{g}$) 25 °C to recover the initial shape. With the temperature continually rising, the shape recovery ratios of all samples rose rapidly. According to Ohm’s law, the total amount of heat (*Q*) produced by Joule heat is written as follows in Equation (3) [37]:

(3) $$Q=I^{2}Rt=\frac{U^{2}}{R}t$$

where *Q* represents the amount of heat produced expressed in Joules, and I is the current in Amperes. *R* is the resistance of the sensor wire offered by the circuit to the electric current (I) flow in Ohms, and *t* is the time when the current is allowed to flow in the circuit, expressed in seconds. Therefore, the smaller the resistance, the greater the heat through the sensor, increasing the recovery rate. Furthermore, during the expansion stage, the breakage of the fragile silver layer results in a large increase in resistance. Recall that, in the formula of the relative resistance changes (∆R/$R_{0}$), the lower the initial resistance ($R_{0}$), the higher the sensitivity. Therefore, the silver layer plays an essential role in making the sensor achieve high sensitivity. In the meantime, the elastic yet moderately conductive bottom layer (SMP-CNTs) temporarily fills the brittle top layer’s prebuilt cracks when released, sustaining the sensor’s conductive networks. The CNTs in the mixture play two important roles: fast recovery rate and stable residual strain value. According to [38], increasing the mass fraction of CNTs inside the SMP composites reduces the shape recovery rate while increasing the stable cyclic stress. However, the high mass fraction of CNTs inside the composite makes the cross-linking network chain dense, leading to the structure of the sensor being more rigid, causing an increase in Young’s modulus and harming the movement of the sensor. In other words, when the mass percentage of the carbon fiber reaches a reasonable level, its shape memory qualities and best mechanical properties are achieved [38]. The sensor was examined for relative resistance changes under various strains (10%, 20%, 25%, and 30%) to show the electrical responses of the sensor (Figure 6 and Figure 7). In this test, the sensor was held for approximately 5 s at a defined tension point before release and repeated five times every 5 s thereafter. As can be seen in Figure 7, the response time is around 200 ms under stretch while the release time is 250 ms. The sensor tends to contract in a direction perpendicular to the elongation stress due to the properties of the polymer, leading to a loosening of the wire when operating under a rapid elongation. This explains why the overshoot in the signal of stretching is higher than at release.

According to Figure 6, the relative variations in the sensor’s resistance are essentially unchanged after one minute of stretching and continuous release, with a change in the acceptable range of less than 2% after ten load cycles. However, mechanical cycling in the long term can lead to wear and tear on the surface of the sensor, which is the silver layer, potentially introducing contaminants or altering the surface properties, which may, in turn, affect the electrical resistance. The measuring factor (GF = (∆R/$R_{0}$)/∆ε)), known as the ratio of the relative change in resistance to mechanical strain, is used to evaluate the sensitivity of the strain sensor, where ∆ε is the mechanical strain applied on the sensor. This value is about 20 at 5% and 8 at 40% in the first stretch. Table 2 compares the parameters of the strain sensor used in our research with those in other references.

### 3.2. Data Collection and Processing and Application of the Respiration Sensor

**Figure 8:**
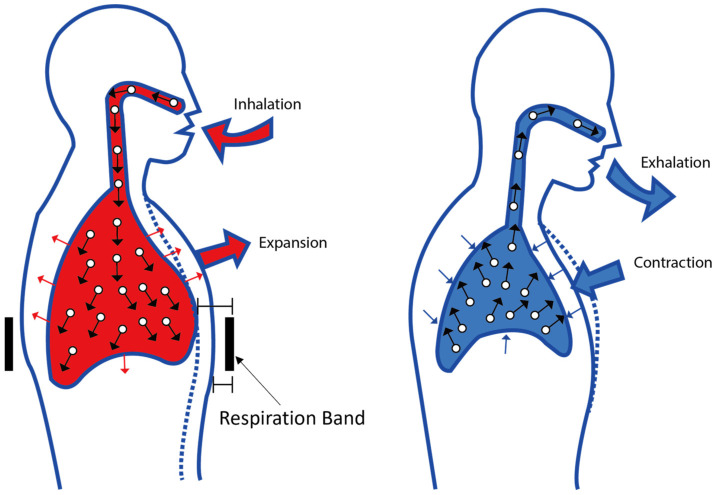
Changes brought on by breathing in the volume of air in the lungs and the size of the abdomen.

In our research, we created a flexible human respiration detector by linking electrodes to a microcontroller and integrating them into bands and clothing. The proposed sensor was employed to monitor human respiration. As established by previous studies, human respiration can be measured from two locations: the chest and the abdominal wall [3,43]. Abdominal breathing is characterized by the expansion of the belly while the diaphragm contracts. In this study, we fabricated a stretchable human respiration detector by connecting electrodes to the microcontroller and assembling them on bands and clothing to demonstrate the potential of silver-coated SWCNT wires in wearable devices. The proposed resistive stretch sensor was applied to measure the respiration of humans. In this study, the entire respiration measurement system was designed as a band worn around the subject’s abdomen, positioned between the rib cage and the abdomen at the level of the umbilicus, as depicted in Figure 8. During physical activity, the contraction of abdominal muscles pushes the diaphragm against the lungs, influencing the respiration band. Consequently, the conductor’s surface area expands as a part of the respiration process, leading to an increase in its electrical resistance. Typically, inspiratory movements of the abdomen and thorax occur nearly simultaneously. However, if there is partial upper airway obstruction, there may be a phase shift and timing differences in the motions of the thorax and abdomen. This can introduce noise into the data collected from the respiration sensor, particularly when the sensor is applied to the band. Careful consideration of these factors is paramount for accurate and reliable respiration monitoring using wearable devices.

**Figure 9:**
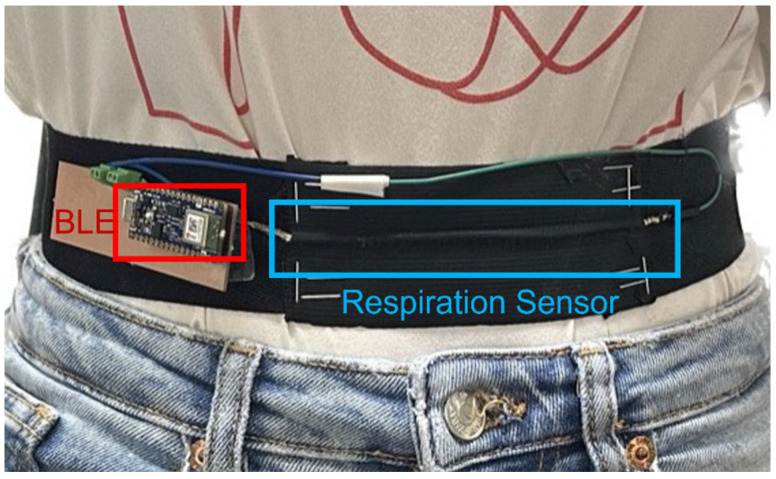
Illustration of respiration band placed on the human body.

The respiration band was constructed using two distinct materials. The section in direct contact with the subject was crafted from a sewing stretch elastic band spool with medium elasticity sourced from Seoul, Republic of Korea. In contrast, the front portion was covered with a polyurethane (PU) film. This choice was made to emphasize the sensor’s stretchability during breathing, with the PU film serving to safeguard the silver coating layer, which is particularly vulnerable and sensitive to stretching, as depicted in Figure 9.

**Figure 10:**
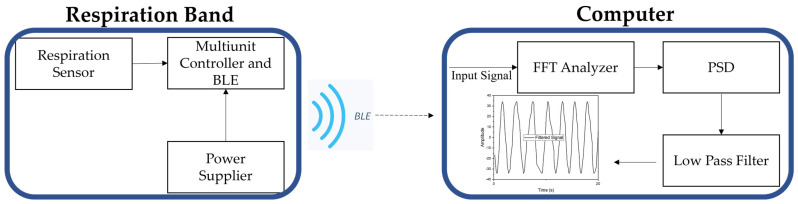
Block diagram of the respiration measuring system.

The system is capable of detecting abdominal breathing vibrations and transmitting them wirelessly through Bluetooth Low Energy (BLE) radio to a computer for data processing, as illustrated in Figure 10. The respiration band is powered by a stable power source, specifically two 3.7 V Li-ion rechargeable batteries connected to a protection board from the Adafruit company, serving as the voltage supplier for the entire band. For the system’s microcontroller unit, we utilized an Arduino Nano BLE board. This board played a crucial role in collecting data from the stretch sensor.

Data obtained from the resistive stretch sensor were transmitted directly to MATLAB (version 2021b) via the embedded Bluetooth Low Energy module in the Arduino board. This setup was designed to streamline the process of visualization and subsequent signal decomposition. The utilization of Bluetooth Low Energy technology not only facilitated efficient wireless data transfer, but also ensured compatibility with the MATLAB platform, allowing for the real-time monitoring and analysis of the obtained sensor data. While the typical respiration rates of an adult at rest range from 12 to 20 breaths per minute, which translates to approximately 0.2 to 0.33 Hz, a sampling rate of 10 Hz was chosen. This decision aimed to comprehensively analyze the sensor’s performance, encompassing any noise that could arise from its structure when applied to the band and the body. It is worth noting that the waist circumference of the body can also contribute to noise during the sensor’s operation.

**Figure 11:**
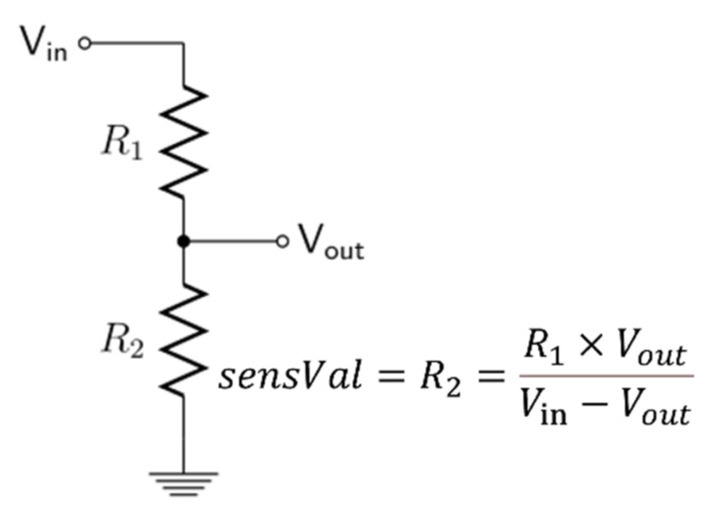
Voltage divider schematics.

To detect the value of the resistive sensor during the object’s breathing, we employed a voltage divider circuit. This simple circuit reduces a higher voltage to a lower one by utilizing two series resistors and an input voltage. Voltage dividers are among the fundamental circuits in electronics. In this case, the analog signal from the sensor depicted in Figure 11 was converted into the resistance value using Equation (4):

(4) $$sensVal=\frac{R_{1}\times V_{out}}{V_{in}-V_{out}}$$

where *sensVal* is the resistance value of the sensor, $R_{1}$ is the reference resistor with the value of 10 kΩ, $V_{in}$ is the voltage that supplies the voltage divider, and $V_{out}$ is the voltage drop on the sensor.

Figure 9 shows the respiration band worn on the abdominal wall of the object. During this experiment, the participant was instructed to perform inhalation and exhalation while at rest for a duration of 120 s while counting their breath rate (as shown in Movie S1). The description of the code for some specific use cases is included in the Supplemental Information. After buffering the data into 120 s windows, we employed fast Fourier transform (FFT) to extract the respiratory rate. The Arduino board collected data at intervals of 100 milliseconds and transmitted it to the computer via BLE. In this setup, the board functioned as a peripheral device, while the computer, equipped with MATLAB software and the Arduino support package, acted as the central device.

Fourier transform is a technique used to analyze the frequency domain characteristics of a signal. The number of discrete frequencies examined in a Fourier transform is directly related to the number of samples in the original waveform. FFT significantly reduces the number of complex computations needed when we assume that N (the length of the signal) is a multiple of 2. This mathematical assumption eliminates redundant calculations and those with no practical value (e.g., multiplication by “1”), resulting in substantial computational efficiency and a reduction in the number of samples required to xog2(N). Consequently, FFT can approximate discrete Fourier transforms much faster, making them a valuable tool for signal analysis.

**Figure 12:**
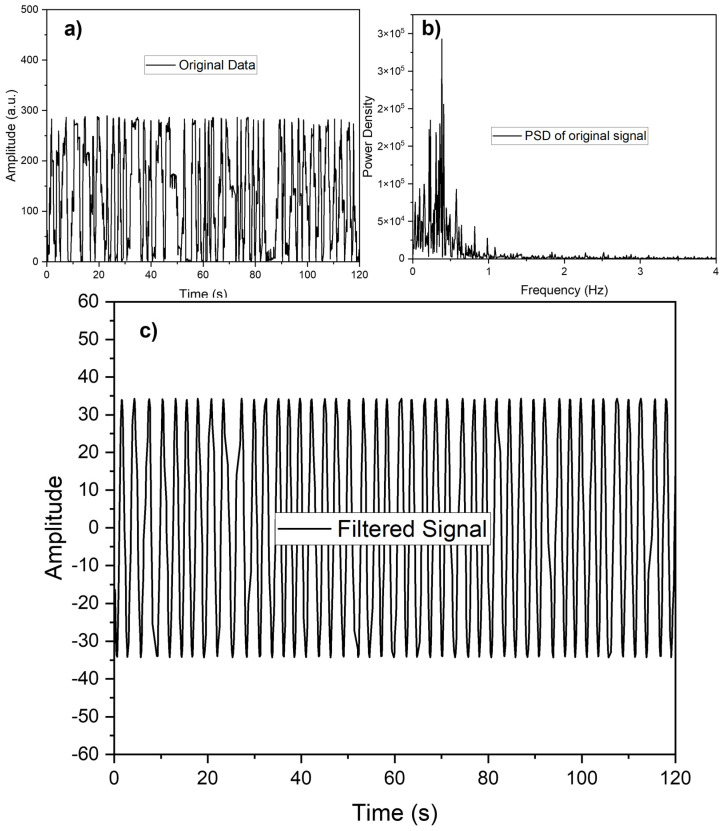
Respiration signals obtained from respiration band. (**a**) Original signal; (**b**) PSD of original signal; (**c**) the signal after filtering.

When applying FFT to explore the frequency domain characteristics of a signal, two key considerations are the detectability of a small signal in the presence of a larger one and frequency resolution, which differentiates between two distinct frequencies. In practice, the signals we measure have finite durations, and the FFT computes the frequency transform over a specific number of discrete frequencies called bins. In this study, the author employed Fourier transform to convert the signal from the time domain to the frequency domain and calculated the power spectrum density (PSD) of each frequency component to gain deeper insights into the data. The data collected by MATLAB had a sampling frequency of approximately 8.3 Hz, as depicted in Figure 12a. Figure 12b illustrates the plot of all frequencies present in the data, with the dominant frequency being around 0.35 Hz. To enhance the visualization of respiration, a filtering process was applied to remove unwanted frequency components, resulting in the output shown in Figure 12c.

## 4. Conclusions

In conclusion, we suggested a quick, simple, and uncomplicated fabrication method for a resistive strain sensor. The strain sensor was created using commercial SMP, silver paste, and CNTs and had high sensing capabilities. The sensor was made up of two layers: a flexible conductive coil (CNT/SMP) and a highly conductive layer (referred to as silver coating). Our sensor had a GF of 20 within 0–5% and displayed high sensitivity. The sensor underwent examination to assess the relative resistance changes across different strains (10%, 20%, 25%, and 30%), illustrating the electrical responses of the sensor. Due to the inclusion of CNTs in the combination, the sensor also exhibited high flexibility, reasonable hysteresis, and high reversibility, stability, and durability. The sensor might also be used on a medium-elasticity sewing stretch elastic band spool in Seoul, Republic of Korea, to detect respiration in humans. The easy, scalable, and quick production method, good performance, and tactile-based characteristics of this strain sensor offer promising benefits for a variety of applications, including the creation of intelligent protective gear. In our ongoing and future research, we focus on exploring stretchable sensing technology integrated with Machine Learning (ML) to develop ML-stretchable electrode systems, emphasizing utilizing these systems for bioelectrical signal recognition to detect abnormal human activity.

## Figures and Tables

**Table 1:** Characteristics of silver paste from KH Chemicals, Korea.

Specification	Property
Curing conditions (°C, min)	120, 10
Density (g/${cm}^{3}$)	>2
Sheet Resistance (mΩ/□/mil)	<30 @120 °C

**Table 2:** Comparison of the proposed sensor with reported strain sensors.

Reference	Principle	Max GF@ Corresponding Strain	Cycles	Response Time (ms)
This work	Resistive	20 @5%	1000	200–250
[39]	Resistive	4.5 @0.6%	1000	240
[40]	Resistive	19 @0.5%	1000	300
[41]	Resistive	8.33 @100%	600	160
[42]	Resistive	30 @100%	2500	-

## Data Availability

The data that support the findings of this study are available from the corresponding author upon reasonable request.

## Supplementary Materials

The following supporting information can be downloaded at: https://www.mdpi.com/article/10.3390/polym16030373/s1, File S1: Coding description; Movie S1: Respiration signals obtained from respiration band.
