# Supplementary material for: A Wearable Strain Sensor Utilizing Shape Memory Polymer/Carbon Nanotube Composites Measuring Respiration Movements
Source: Polymers (Basel). 2024 Jan 29;16(3):373. doi: 10.3390/polym16030373 (PMC10857196; doi:10.3390/polym16030373)
Supplement: Supplementary file 1 [file polymers-16-00373-s001.zip › File S1.pdf]

Supplementary Information

A Wearable Strain Sensor Utilizing Shape Memory Polymer/Carbon Nanotube Composites Measuring Respiration Movements

TranThuyNga Truong [1] and Jooyong Kim *

* Department of Materials Science and Engineering, Soongsil University, Seoul 156-743, Korea

[1]Department of Smart Wearables Engineering, Soongsil University, Seoul 156-743, Korea

MATLAB description

This code is a MATLAB script responsible for connecting to a Bluetooth Low Energy (BLE) device, collecting sensor data, and performing signal processing and visualization tasks. Below is an explanation of the key sections and functions in the code:

BLE Connection:

- **`ble("BreathRateMeasurement")`:** It connects to a specific BLE device with the name "BreathRateMeasurement."
- **`characteristic(b, "3a40fc76-3646-474c-8fbc-100fab49cc3e", "0be59839-c6d9-4e2c-a020-adc952d6393e")`:** It specifies a specific characteristic on the BLE device using its UUID (Universally Unique Identifier).

Data Acquisition:

xVal, data, and t are initialized as empty arrays to store sensor data and timestamps.

- **`subscribe(c)`:** This function subscribes to notifications from the specified BLE characteristic.

Data Collection Loop:

- time = 120: A time limit for data collection is set to 120 seconds.
- Inside the for loop (executed only once), sensor data is continuously collected for a duration of 120 seconds. The **`displayCharacteristicData`** function reads and processes data from the BLE characteristic.

Signal Processing and Visualization:

- The collected data is plotted as an original signal in Figure 1. Signal processing tasks include detrending, computing the power spectral density (PSD), and performing a Fast Fourier Transform (FFT) to analyze the data.
- The results are plotted and saved as figures and data files.

Filtering:

- The code performs filtering on the data. It applies a low-pass filter (lowpass) to data with a cutoff frequency of 0.5 Hz. The filtered signal is then plotted.

Additional Processing (Optional):

- A section calculates and displays the filtered signal after further processing, which may be related to SampEn (Sample Entropy).
- **`displayCharacteristicData` Function:** This function reads data from the BLE characteristic and converts it into a numeric value for further processing. It appears to handle the reverse byte order and hex-to-decimal conversion.

Unsubscribe:

- **`unsubscribe(c)`:** This function unsubscribes from notifications from the BLE characteristic, ending the data collection.

Arduino description

The provided Arduino code is for a Bluetooth Low Energy (BLE) peripheral device. This device measures the resistance of a sensor connected to analog input A0 and broadcasts this data via BLE. Here's a breakdown of the code:

Include Libraries:

- The code starts by including the ArduinoBLE library, which provides functions for setting up the BLE peripheral.

Define Data Structures:

- The code defines a data structure to hold the sensor data read from analog input A0. It specifies a floating-point value for A0.
- A union is used to map this data structure to a byte array, which is important for sending data over BLE.

Global Variables:

- **`previousMillis`:** A variable to keep track of the time for sending data updates.
- **`raw0`:** Stores the last recorded analog reading from A0.

Device and Service Configuration:

- The device name, service UUID, and characteristic UUIDs are defined.
- A BLE service (**`BRMService`**) and a characteristic (**`A0Char`**) are created.

setup() Function:

- Serial communication is initialized.

- The **LED_BUILTIN** pin is configured as an output (presumably to indicate the device's status).
- BLE is initialized, and if it fails to start, the program enters an error loop.
- The local name and advertised service UUID are set.
- The characteristic is added to the service, and the service is added to the BLE stack.
- The device is set to advertise its presence.

loop() Function:

- The main loop waits for a central (another BLE device) to connect to the peripheral.
- When a central device connects, it is identified by its address.
- The **LED_BUILTIN** is turned on to indicate the connection.
- While the central device is connected, the code enters a loop where it periodically updates and sends data from the A0 sensor.

updateValue() Function:

- The function reads the analog value from A0.
- It calculates the resistance based on the sensor configuration (R1 and Vin).
- The calculated resistance is stored in the **'A0Data'** structure.
- The code checks if the analog reading has changed and if so, it sends the updated data via BLE by calling **'A0Char.writeValue'**.
